# RASSF1A inhibits gastric cancer cell proliferation by miR-711- mediated downregulation of CDK4 expression

**DOI:** 10.18632/oncotarget.6813

**Published:** 2016-01-02

**Authors:** Aijun Liao, Gao Tan, Lin Chen, Weiwei Zhou, Hongsai Hu

**Affiliations:** ^1^ Department of Gastroenterology, The First Affiliated Hospital of South China University, Hengyang, Hunan Province, China; ^2^ Gastric Cancer Research Center of Hunan Province, Hunan, China; ^3^ Guangdong Provincial Key Laboratory of Gastroenterology, Department of Gastroenterology, Nanfang Hospital, Southern Medical University, Guangzhou, Guangdong Province, China

**Keywords:** RASSF1A, miR-711, CDK4, gastric cancer, cell cycle

## Abstract

Although interaction with DNA repair proteins has demonstrated that RASSF1A is a tumour suppressor gene, much attention has been directed in recent years towards its roles in regulating the cell cycle. However, the precise mechanism remains unclear. Uncovering how RASSF1A participates in regulating the cell cycle is critical to exploring effective therapeutic targets for gastric cancer. Here we show that RASSF1A could regulate 14 miRNAs’ expression in the typical human gastric cancer line SGC-7901, of which miR-711 was upregulated the most. Moreover, for SGC-7901 cells, miR-711 was found to downregulate CDK4 expression, and to arrest the cell cycle in the G1 phase. Our results suggest that RASSF1A inhibits the proliferation of gastric cancer cells by upregulating the expression of miR-711, which arrested gastric cancer cells in the G1 phase by downregulating expression of CDK4. This finding might provide us with a novel therapeutic target for gastric cancer by increasing RASSF1A expression via miR-711 regulation.

## INTRODUCTION

Gastric cancer remains a major public health problem and one of the major contributors to cancer-related deaths around the world[1–3]. As with most human cancers, gastric carcinogenesis is a complex multistep process involving the activation of proto-oncogenes and/or the inactivation of tumour-suppressor genes[4]. One such tumour-suppressor gene, Ras association domain family protein 1 isoform A (RASSF1A) [5], is frequently lost or is expressed at reduced levels in gastric cancer cells[6]. Molecularly, *RASSF1A* is localized at chromosome 3p21.3, a locus that shows frequent loss of heterozygosity in gastric cancer[6]or that is silenced due to gene promoter hypermethylation[7]. The RASSF1A protein plays important roles in regulating cell cycle progression, apoptosis, and microtubule stability[8]. However, the precise molecular mechanism of the antitumour activity of RASSF1A remains to be elucidated.

Although gastric carcinogenesis is involved in the genetic dysregulation of proto-oncogenes and tumour suppressor genes, many recent discoveries have shed new light on the involvement of microRNAs (miRNAs) in gastric cancer[9–11]. miRNAs are small noncoding RNA molecules in cells and tissues that can post-transcriptionally regulate gene expression[12, 13]. Therefore, they are involved in diverse crucial biological functions, such as development, proliferation, differentiation and apoptosis[14, 15]. A large number of studies have demonstrated that aberrant expression of miRNAs is associated with human diseases, such as cancer. Depending on the target genes, miRNAs can function as proto-oncogenes and tumour-suppressor genes[16]. A significant number of miRNAs have been mapped to cancer-associated genomic regions. To date, miR-17, miR-18a\b, miR-19a, miR-20a\b, miR-21, miR-106a\b, miR-340, miR-421, and miR-658 have been shown to be highly expressed in gastric cancer tissues[17–20], whereas the expression of miR-34b, miR-34c, and miR-128a is upregulated in undifferentiated gastric cancer tissues[21]. In contrast, the expression of miR-128b, miR-129 and miR-148 is downregulated in gastric cancer tissues[22].

The purpose of this study was to determine whether RASSF1A inhibited gastric cancer cell activities by regulating the expression of relative miRNAs. For this purpose, we used the typical human gastric cancer line SGC-7901 to investigate the underlying mechanism.

## RESULTS

### Inhibition of the viability, migration and invasion capacity of SGC-7901 cells by RASSF1A

**Figure 1:**
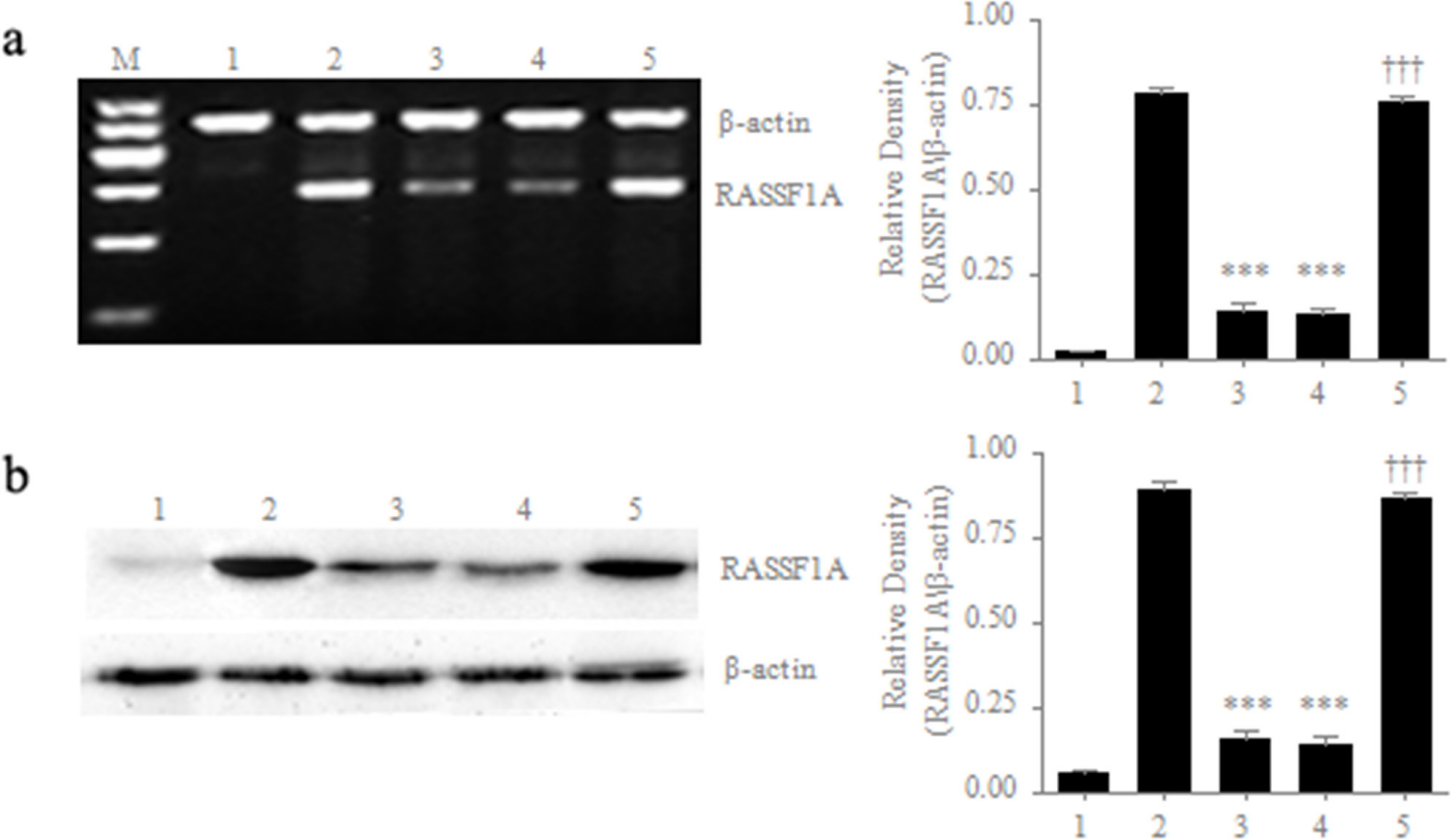
Differential expression of RASSF1A in different cell lines mRNA **a.** and protein **b.** levels of RASSF1A expression were determined by RT-PCR and western blot, respectively. M: marker; 1: Negative control of HepG2 cells; 2: Positive control of normal gastric mucosal cells; 3: Parental SGC-7901 cells; 4: SGC-7901 cells transfected with pcDNA3.1 plasmid; 5: SGC-7901 cells transfected with pcDNA3.1-RASSF1A. ***P< 0.001 vs. 2 group; ***P < 0.001 vs. 4 group. The data are shown as the means ± SDs of three independent experiments.

In order to assess the effects of RASSF1A on the regulation of the biological activities of gastric cancer cells, we first established stable RASSF1A-expressing gastric cancer cells, because RASSF1A is frequently lost or is expressed at low levels in gastric cancer cells. We stably transfected SGC-7901 cells (a typical cell line of human gastric carcinoma) with RASSF1A cDNA and then determined the mRNA and protein levels of RASSF1A expression by RT-PCR and Western blotting, respectively. We found that the mRNA levels of RASSF1A expression were 5.85-fold higher in the pcDNA3.1-RASSF1A-transfected cells than in the pcDNA3.1-vector-transfected cells (*P* < 0.001; Figure 1a). However, there was no significant difference in mRNA levels between the parental SGC-7901 cells and the pcDNA3.1-vector-transfected SGC-7901 cells (*P* = 0.469; Figure 1a). Similarly, we also found that the protein levels of RASSF1A expression were 6.14-fold higher in the pcDNA3.1-RASSF1A-transfected cells than in the pcDNA3.1-vector-transfected cells (*P* < 0.001; Figure 1b). However, there was no significant difference in protein levels between the parental SGC-7901 cells and the pcDNA3.1-vector-transfected SGC-7901 cells (*P* = 0.374; Figure 1b).

**Figure 2:**
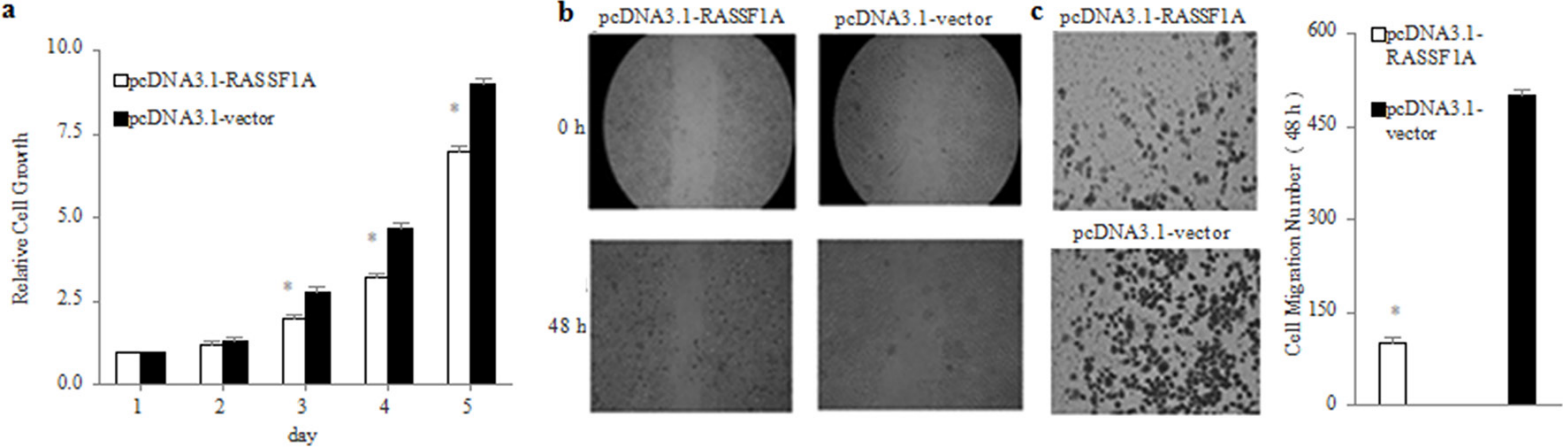
Inhibition of viability, migration and invasion of SGC-7901 cells by RASSF1A *in vitro* Viability **a.**, migration **b.** and invasion **c.** of SGC-7901 cells were determined by MTT assay, Wound healing assay and transwell invasion assay at the indicated time points, respectively. *P< 0.05 vs. pcDNA3.1-vector-transfected cells. The data are shown as the means ± SDs of three independent experiments.

After the establishment of stable RASSF1A-expressing gastric cancer cells, we next assessed the effects of RASSF1A on the regulation of SGC-7901 viable cells, migration and invasion *in vitro* by MTT assay, wound healing assay and transwell tumour cell invasion assay, respectively. We found that the SGC-7901 viable cells in culture was increasingly lower from 2 to 5 days in the pcDNA3.1-RASSF1A-transfected cells than in the pcDNA3.1-vector-transfected cells (*P* < 0.05; Figure 2a). In addition, we found that the migration capacity of the pcDNA3.1-RASSF1A-transfected cells was more significantly decreased at 0 h and 48 h than that of the pcDNA3.1-vector-transfected cells (*P* < 0.05; Figure 2b). Furthermore, we found that the number of invading cells was significantly fewer in the pcDNA3.1-RASSF1A-transfected cells than in the pcDNA3.1-vector-transfected cells (*P* < 0.05; Figure 2c). Therefore, these results clearly indicated that the expression of RASSF1A could suppress the viability, migration and invasion capacity of SGC-7901 cell *in vitro*.

### Effect of RASSF1A on the regulation of miRNA expression

Although RASSF1A could suppress the viability, migration and invasion capacity of SGC-7901 cells, the mechanism remains to be elucidated. Because miRNAs are involved in diverse biological functions, such as cell proliferation and differentiation and play important roles in transcriptional control of gene expression[23], we determined using miRNA microarray whether RASSF1A could regulate the expression of miRNAs to inhibit the activities of SGC-7901 cells. We found that there were 14 differentially expressed miRNAs, half of which were upregulated and the other half of which were downregulated in RASSF1A-overexpressing SGC-7901 cells (Table S1).

**Figure 3:**
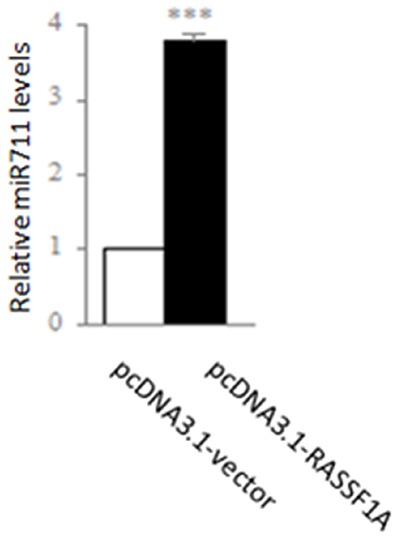
Regulation of expression of miR-711 by RASSF1A in the SGC-7901 gastric cancer cell line SGC-7901 cells were transfected with pcDNA3.1-vector or pcDNA3.1-RASSF1A and, then the expression was determined of miR-711 by qPCR. **P< 0.01, ***P< 0.001 vs. pcDNA3.1-vector-transfected cells. The data are shown as the means ± SDs of three independent experiments.

Because the levels of miR-711 expression were most significantly regulated by RASSF1A in the 14 miRNAs (Figure S2), we subsequently substantiated that RASSF1A could regulate the expression of these miRNAs by qRT-PCR. We found that the expression of miR-711 was 3.78-fold higher in the pcDNA3.1-RASSF1A-transfected SGC-7901 cells than in the pcDNA3.1-vector-transfected SGC-7901 cells (miR-711, *P* < 0.001; Figure 3). These results suggested that RASSF1A could inhibit the activities of SGC-7901 cells by regulating the expression of these miRNAs.

### Differential expression of miR-711 and RASSF1A in gastric cancer tissues

**Figure 4:**
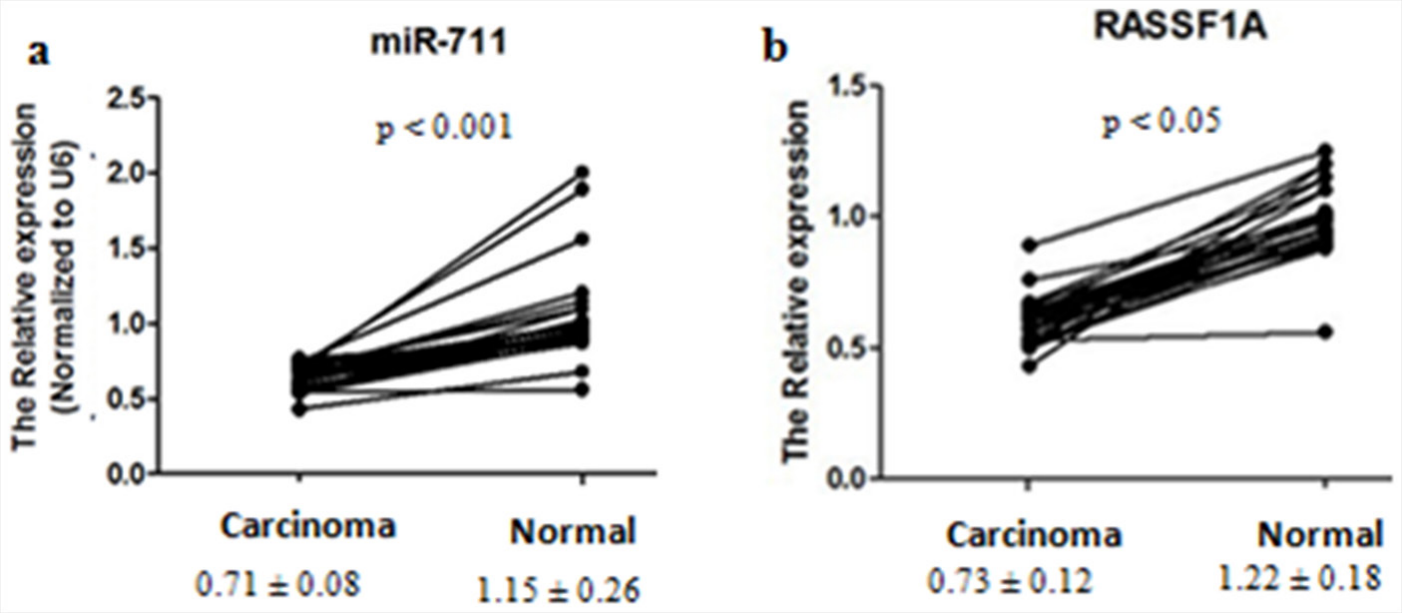
Differential expression of miR-711 and RASSF1A between gastric cancer tissues and normal gastric mucosa tissues Gastric cancer tissues and the corresponding normal gastric mucosa tissues were collected from 28 patients. Total RNA was isolated, and mRNA expression of miR-711 **a.**, and RASSF1A **b.** was determined by real-time PCR. The means (± SDs) levels of miR-711 and RASSF1A in gastric cancer tissues and normal gastric mucosa tissues are shown under the x-axis.

To confirm further that miR-711 and RASSF1A were differentially expressed between gastric cancer tissues and normal gastric mucosa tissues, we compared the expression between the gastric cancer tissues and the corresponding normal tissues by qRT-PCR. We found that the expression of miR-711 and RASSF1A mRNA was 0.62-fold and 0.60-fold lower in the cancer tissues than in the normal tissues, respectively (Figure 4). Therefore, these results not only showed that miR-711 and RASSF1A were differentially expressed between gastric cancer tissues and normal gastric tissues, but they further confirmed the above results of miRNA microarray.

### Inhibition of the viability of SGC-7901 cells by miR-711-mediated downregulation of CDK4 expression

**Figure 5:**
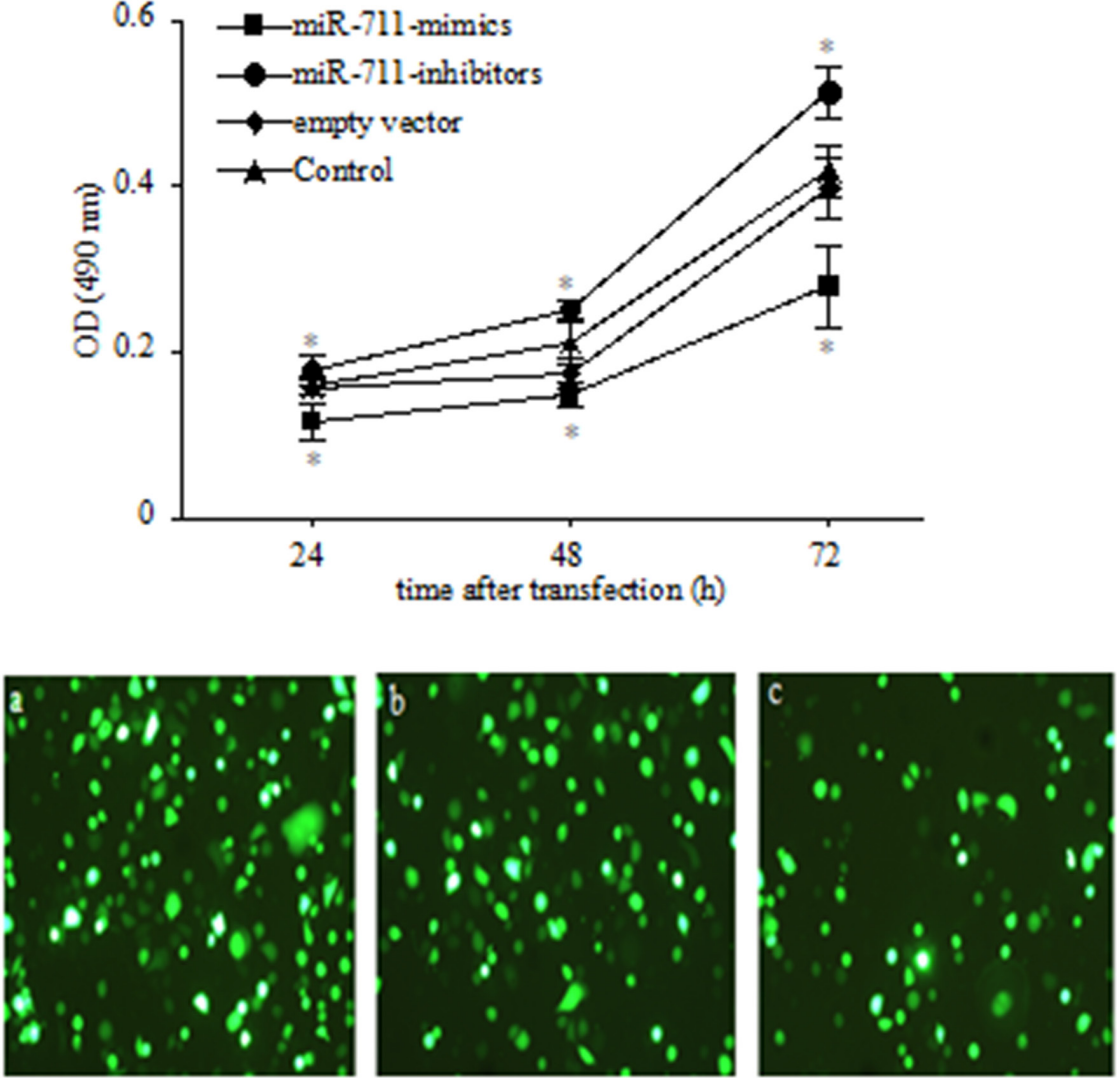
Inhibition of SGC-7901 viable cells by miR-711 **Top**: SGC-7901 cells were transfected with miR-711 mimics, miR-711-inhibitors and empty vector. Cells were collected at the indicated time points, and viable cells was determined by MTT assay. **Bottom**: transfection efficiency with GFP-labelled vector in SGC-7901 cells. SGC-7901 cells were transfected with miR-711-mimics **a.**, miR-711-inhibitors **b.** and empty vector **c.** using a 1:250 dilution of Lipofectamine 2000 for 4 h. After 18 h, the transfection efficiency was checked with fluorescence microscopy, and the cells were used for experiments. *P < 0.05 vs. untransfected control group. The data are shown as the means ± SDs of three independent experiments.

Because the levels of miR-711 expression were most significantly regulated by RASSF1A in the 14 miRNAs, we next determined the role of miR-711 in regulating the growth of SGC-7901 cells. We assessed by MTT assay the viability of SGC-7901 cells transfected with miR-711-mimics, miR-711-inhibitors or empty vector 24 h, 48 h and 72 h after transfection. We found that the SGC-7901 viable cells was increasingly lower from 24 h to 72 h in the miR-711-mimic-transfected cells than in the untransfected cells (control) (*P* < 0.05; Figure 5 Top). However, the SGC-7901 viable cells was increasingly higher from 24 h to 72 h in the miR-711-inhibitor-transfected cells than in the controls (*P* < 0.05; Figure 5 Top). These results indicated that miR-711 could suppress SGC-7901 cell growth in a time-dependent manner.

**Figure 6:**
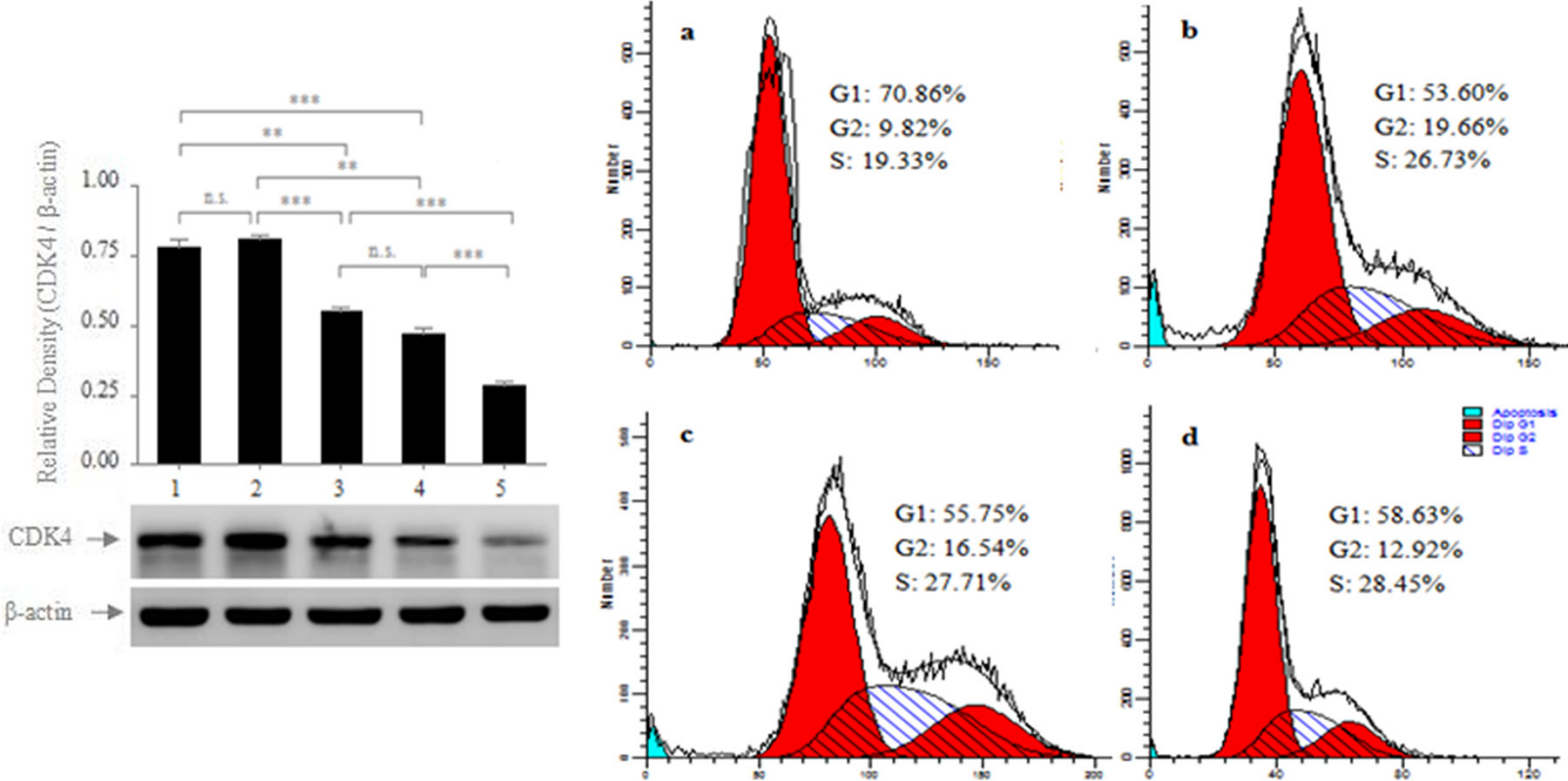
Regulation of the protein expression of CDK4 by miR-711 **Left**: RASSF1A-overexpressed SGC-7901 cells were transfected with miR-711-inhibitors (1); SGC-7901 cells were transfected with miR-711-inhibitors (2), empty vector (3); SGC-7901 cells were not transfected (4); SGC-7901 cells were transfected with miR-711 mimics (5). After 72 h of transfection, the whole-cell extracts were prepared and were analysed for CDK4 and β-actin by western blotting. **Right**: Effect of miR-711 on the cell cycle of SGC-7901 cells. SGC-7901 cells were transfected with miR-711 mimics **a.**, miR-711-inhibitors **b.** and empty vector **c.** After 72 h of transfection, the cell cycles of the transfected cells (a, b and c) and the untransfected cells **d.** were analysed by flow cytometry. The results are representative of three independent experiments. n.s., not significant, **P< 0.01; ***P< 0.001.

Because cyclins and cyclins-dependent kinases (CDKs) are vital factors in the regulation of the cell cycle, and they play important roles in the pathogenesis and development of gastric cancer[24, 25] and because many miRNAs play crucial roles in regulating the cell cycle[26, 27], we determined whether miR-711 suppressed SGC-7901 cell growth by regulating the expression of relative cyclins and CDK. Because preliminary bioinformatics analysis and a pull-down assay revealed that CDK4 was the precise biological target gene of miR-711 (Figure S1), we determined whether miR-711 could regulate CDK4 expression. We found that CDK4 protein expression was lower in the miR-711-mimic-transfected SGC-7901cells but higher in the miR-711-inhibitor-transfected SGC-7901cells and RASSF1A-overexpressed SGC-7901 cells transfected with miR-711-inhibitors, compared with the empty vector-transfected cells and untransfected cells (Figure 6 Left). These results suggested that miR-711 could downregulate CDK4 expression to inhibit the viability of SGC-7901 cells.

Because CDK4 plays important roles in regulating the cell cycle from the G1 to S phase[28, 29], we subsequently determined whether miR-711 inhibited cell proliferation by affecting the cell cycle. We transfected the SGC-7901 cells with miR-711-mimics, miR-711-mimics and empty vector, and we assayed the cell cycle 72 h after transfection. We found that the miR-711-mimic-transfected cells were arrested in the G1 phase more than the empty vector-transfected cells or untransfected cells (Figure 6 Right). This result provided evidence that miR-711-mediated downregulation of CDK4 expression inhibited cell proliferation by arresting the cell cycle in the G1 phase.

## DISCUSSION

This study focused on the mechanism by which RASSF1A participated in the process of gastric carcinogenesis and development. Our results showed that RASSF1A could downregulate gastric cancer activities, possibly by modulating relative miRNAs. In the typical gastric cell line SGC-7901, we found that the capacities for viability, migration and invasion were all significantly decreased in the RASSF1A-transfected cells, compared with the vector-transfected cells (Figure 2). This finding was in agreement with previous findings showing that RASSF1A proteins possess important anti-tumour activities[30–32].

In further mechanism studies, our data showed that RASSF1A modulated the expression of 14 miRNAs, half of which were upregulated and the other half of which were downregulated in the RASSF1A-overexpressing SGC-7901 cells (Table S2). Regarding the most relative miRNA to RASSF1A, RASSF1A was positively correlated with miR-711 expression. We found that the expression of miR-711 expression was significantly higher in the RASSF1A-transfected cells, compared with the vector-transfected cells (Figure 3). In addition, we also found that the expression of miR-711 and RASSF1A was significantly lower in the gastric cancer tissues, compared with normal gastric tissues (Figure 4).

Although the levels of miR-711 expression were most significantly regulated by RASSF1A in the 14 miRNAs, it remains unclear whether RASSF1A participated in regulating the viability of SGC-7901 cells by miR-711. However, our data demonstrated that miR-711 could suppress SGC-7901 cell growth by downregulating CDK4 expression. We found that the viable cells and protein expression of CDK4 were both significantly decreased in the miR-711-mimic-transfected cells but were significantly increased in the miR-711-inhibitor-transfected cells, compared with untransfected SGC-7901 cells (Figures 5 and 6). Therefore, our findings revealed that RASSF1A inhibited the viability of SGC-7901 cells by miR-711-mediated downregulation of CDK4 expression. Together, these results indicated that miR-711 could inhibit human gastric cancer viable cells.

As discussed above, our findings suggested that miR-711 might be negatively correlated with human gastric cancer viable cells. This finding relating to miR-711-mediated inhibition of viable cells was in agreement with a previous study indicating that miR-711 could target and suppress the expression of heat shock protein 70.3, which possesses general cytoprotective properties in protecting cells against stressful and noxious stimuli[33]. However, miR-711 could be induced in cutaneous T-cell lymphoma[34]. This result suggested that miR-711 might be positively correlated with viable cells. Therefore, miR-711 might play different roles under various growth conditions, and its role in regulating viable cells remains to be studied further.

It has been suggested that miRNAs inhibit the protein expression of target genes mainly by imperfect base-pairing with the 3′ untranslated region of target mRNAs[35]. However, it is unclear whether miR-711 inhibited the protein expression of CDK4 in this manner or by regulating the expression of upstream genes. Therefore, it would be extremely interesting to explore the mechanism by which miR-711 regulates the protein expression of CDK4.

In summary, this study showed that RASSF1A could suppress the viability of SGC-7901 cells by miR-711-mediated downregulation of CDK4 expression. Although previous studies have shown that RASSF1A can function as a tumour suppressor gene by interacting with DNA repair proteins[30, 36], we showed that RASSF1A could inhibit the growth of gastric cancer cells by upregulating the expression of miR-711, which arrests gastric cancer cells in the G1 phase of the cell cycle by downregulating the expression of CDK4. This finding is significant, because it not only could offer us a new view on RASSF1A as a tumour suppressor gene by regulating miRNA expression, but it might also provide us with a novel therapeutic target for gastric cancer by increasing RASSF1A expression via miR-711 regulation.

## MATERIALS AND METHODS

### Cell culture and transfection

The human gastric cancer cell line SGC-7901 was obtained from Shanghai Cell Bank, Chinese Academy of Sciences (Shanghai, China), and was cultured in RPMI 1640 medium (Life Technologies Inc., Grand Island, NY, USA) supplemented with 10% foetal bovine serum plus penicillin (50 IU/ml) and streptomycin (50 μg/ml) in a humidified incubator containing 5 ml/L CO2 at 37°C. The growth medium was refreshed every 2 days.

To manipulate RASSF1A expression in gastric cancer cells, we first cloned RASSF1A cDNA into a pCDA3.1 vector at the *Bam*HI and *Xho*I sites. After DNA sequence confirmation, the pcDNA3.1-RASSF1A and control pcDNA3.1-vector were stably transfected into the SGC-7901 human gastric carcinoma cell line using Lipofectamine 2000 reagent (Invitrogen, Carlsbad, CA, USA), according to the manufacturer's instructions, and SGC-7901 cells stably expressing the RASSF1A gene were established by G418 selection.

To determine the role of miR-711 in regulating the growth of SGC-7901 cells, we transfected SGC-7901 cells with the miR-711 overexpression vector (miR-711-mimics), miR-711 expression-inhibiting vector (miR-711-inhibitors) or empty vector (NC) (GenePharma Co., Ltd, Shanghai, China) and then assayed the viability of SGC-7901 cells by MTT 24 h, 48 h and 72 h after transfection. Transfection efficiency was tested at 24 h with GFP-labelled vector (Figure 5 Bottom).

### RNA isolation and miRNA microarray profiling

Total cellular RNA was isolated from cell culture using TRIzol® reagent (Invitrogen) and was purified with an RNeasy mini kit (Qiagen), according to the manufacturers’ instructions. The quantity and quality of these RNA samples were estimated using a Nanodrop ND-100 spectrophotometer (Thermo Scientific, USA). Only samples with a 260/280 absorbance ratio > 1.8 were used in this study. Then, these RNA samples were labelled with the miRCURY™ Hy3™/Hy5™ Power labelling kit and were hybridized onto the miRCURY™ LNA Array containing 3100 kinds of probes (version 14.0) using a hybridization station. The data were scanned with the Axon GenePix 4000B microarray scanner, and GenePix pro software, version 6.0, was used to read the raw intensity of the image data.

### Analysis of differential miRNA expression in gastric cancer cells

The miRNA microarray profiling data were analysed using a threshold value of fold change ≥ 2.0 or ≤0.5 (*P* ≤0.05). The heat map diagram shows the results of the two-way hierarchical clustering of genes and samples (Figure S2). Each row represents a miRNA, and each column represents a sample. The miRNA-clustering tree is shown on the left, and the sample-clustering tree appears at the top. The colour scale shown at the top illustrates the relative expression level of a miRNA: red represents a high expression level, and green represents a low expression level.

### Viable cells MTT assay

To detect changes in viable cells after RASSF1A transfection, the MTT (3-(4,5-dimethylthiazol-2-yl)-2,5-diphenyltetrazolium bromide) assay was performed. In brief, at 24 h after gene transfection, cells were digested with 0.25% trypsin, and their concentration was adjusted to 5×10_4_/ml in a single-cell suspension before seeding into 96-well plates at 100 μl/well for culture for up to 5 days. At the end of each experiment, the MTT assay was performed to analyse viable cells, and the experimental results were plotted as a chart of means ± standard deviations, according to a previous study.

### Wound healing assay

After transfection with plasmid pcDNA3.1-RASSF1A or pcDNA3.1, cells were grown to 90% confluence in 6-well dishes. A wound was then created using a sterile 10-μl pipette tip, followed by washing with phosphate-buffered saline (PBS) to remove cell debris. Then, the cells were further cultured in a medium with 5% serum, and the migration rate at the corresponding wound site was documented using a Nikon inverted microscope (Nikon, Japan) at different time points (0 h and 48 h). The wound distance was measured, and the means and standard deviations for transfected cells were determined.

### Transwell tumour cell invasion assay

To assess tumour cell invasion capacity, we performed a transwell cell invasion assay. Briefly, 25 μl of BD Matrigel were added to each upper chamber of transwell plates, which were placed in a 37°C incubator for 2-3 h for the solidification of the Matrigel. Cells after 24 h transfection were trypsinized and adjusted to 8×10_5_ cells/ml in suspension. Then, 200 μl of cell solution were added to the upper chamber of each transwell, and 800 μl of growth medium with 20% FBS were added to the lower chamber. The cells were then cultured at 37°C for 48 h. Next, the cells on the surface of the upper chamber were swabbed with a cotton swab, and the cells under the surface in the lower chamber were stained with crystal violet (0.1%). The cells were then photographed under an inverted microscope and counted to assess cell invasion.

### Quantitative reverse transcription-polymerase chain reaction (qRT-PCR)

Total cellular RNA was extracted using TRIzol® reagent (Invitrogen). The primers for RT–PCR to detect miRNA were designed based on the miRNA sequences provided by the Sanger Center miRNA Registry. The primers (Table S2) were synthesized and purified by Shanghai Gene-Pharma Co. (Shanghai, China). RT reactions were performed using the iScript cDNA synthesis kit (Bio-Rad, Hercules, CA, USA), and qPCR was performed on the Bio-Rad iQTM5 Multicolour Real-Time PCR Detection System (Bio-Rad). The qPCR cycle was 98°C for 2 min, followed by 40 cycles of 95°C for 15 s and 60°C for 30 s, and a final melt-curve analysis (60–95°C) was included. The standard curve was produced with slopes of approximately −3.32 (approximately 100% efficiency); miRNA PCR quantification was performed using the 2ΔΔct method, against U6 for normalization. mRNA PCR quantification used the 2ΔΔct method against β-actin for normalization. The data are the mean values of three experiments.

### RNA pull-down assay

For assays using lysates of SGC-7901 cells containing 3 mg of proteins were prepared, and incubated with 8 nmol of biotin fusion peptides and 40 μl of Streptavidin-Sepharose. After 2 h of incubation at 4°C, beads were washed three times with lysis buffer. Pulled-down proteins were eluted with SDS-PAGE sample buffer (10 mM Tris, pH 7.8, 3% SDS, 5% glycerol, 0.02% bromophenol blue and 2% 2-mercaptoethanol), and analyzed by western blot.

### Protein extraction and western blot

Cells were lysed for 30 min on ice in RIPA lysis buffer (10 mM Tris [pH 8.0], 150 mM NaCl, 1% Nonidet P-40, 0.1% SDS, and 0.5% deoxycholate), supplemented with a protease inhibitor PMSF, and they were centrifuged at 14,000 × *g* for 30 min at 4°C and the supernatants collected. SDS-polyacrylamide gel electrophoresis and western blotting were performed in accordance with standard protocols. Antibodies to mouse monoclonal antibodies RASSF1A and CDK4 (Abcam, Cambridge, UK) and rabbit polyclonal antibody β-actin (Boshide, Wuhan, China) were all diluted at 1:1000. Secondary antibodies were all diluted at 1:4000. Image J software was used to quantify and analyze the density of the protein bands.

### Gastric cancer tissue specimens

To verify differentially expressed genes in gastric cancer, we collected tissue samples from the gastric mucosa of 28 patients with gastric cancer and the corresponding normal gastric mucosa tissues. This research was approved by the Ethics Committee at the First Affiliated Hospital of South China University, and informed consent was obtained from the subjects or their guardians, and the methods were carried out in “accordance” with the approved guidelines.

### Cell cycle analysis by flow cytometry

At 72 h after transfection with miR-711 mimics, miR-711-inhibitors and empty vector, these transfected and untransfected control cells were trypsinized, resuspended in PBS and then fixed in cold ethanol at 4°C overnight. Subsequently, these treated cells were stained with 40 μg/ml propidium iodide and 100 μg/ml RNase A in PBS for 30 minutes at 37°C. Finally, the cell cycle was analysed with a BD Biosciences FACSCalibur flow cytometer.

### Statistical analyses

The data are presented as the means and standard deviations. Statistical significance of comparison between two groups was determined by Student's *t-*test; Statistical significance of multiple comparisons was determined by one-way analysis of variance with Tukey's multiple comparisons under equal variances or with Dunnett T3's multiple comparisons under unequal variances. Statistical significance of gene expression between normal and cancerous tissues was determined by Spearman's test. The statistical analyses were performed using SPSS (Statistical Package for the Social Sciences) software, version 11.0 (SPSS, Chicago, IL, USA). All *p* values were analysed as two-sided, and a value of *P* < 0.05 was considered to be statistically significant.

## SUPPLEMENTARY FIGURES AND TABLES


