# Supplementary material for: RASSF1A inhibits gastric cancer cell proliferation by miR-711- mediated downregulation of CDK4 expression
Source: Oncotarget. 2016 Jan 2;7(5):5842–51. doi: 10.18632/oncotarget.6813 (PMC4868725; doi:10.18632/oncotarget.6813)
Supplement: Supplementary file 1 [file oncotarget-07-5842-s001.pdf]

SUPPLEMENTARY FIGURES AND TABLES

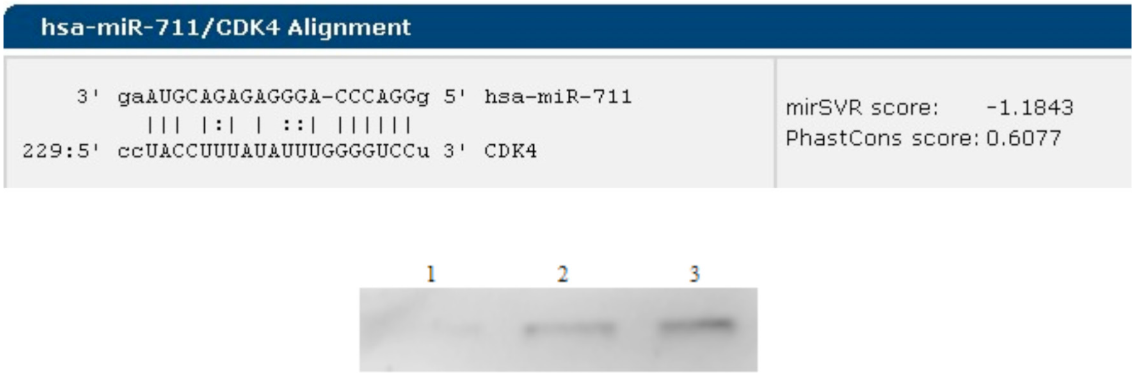

Supplementary Figure S1: Determination of target genes of miR-711 by microRNA (http://www.microRNA.org/). Top: microRNA predicts biological target genes of miRNAs by searching for the presence of conserved 8mer and 7mer sites that match the seed region of each miRNA. We found that CDK4 was the precise biological target gene of miR-711 by microRNA. **Bottom**: A pull-down assay was performed using SGC-7901 cell lysate.1 represents negative control using GAPDH RNA; 2 represents positive control using total cell lysates; 3 represents experimental group using miR-711 RNA and the RNA-pulled down protein.

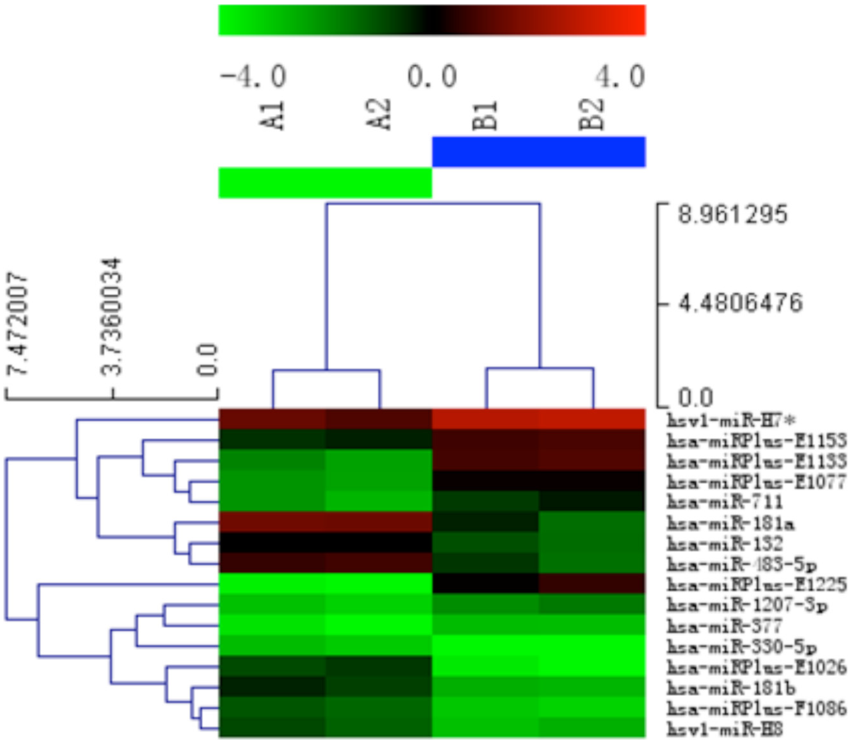

Supplementary Figure S2: miRNA microarray profiling of differentially expressed miRNAs between RASSF1A-expressing cells and control cells. The heat map diagram shows the results of the two-way hierarchical clustering of genes and samples. Each row represents a miRNA, and each column represents a sample. The miRNA-clustering tree is shown on the left, and the sample-clustering tree appears at the top. The colour scale shown at the top illustrates the relative expression level of a miRNA. Red represents upregulated miRNA expression, and green represents downregulated miRNA expression.

Supplementary Table S1: Regulation of miRNA expression by RASSF1A in SGC-7901 cells

ID	Name	Fold change	*p*
46555	hsa-miRPlus-E1153	2.488230252	0.006606526
46885	hsa-miRPlus-E1225	24.05928045	0.031592253
46383	hsa-miRPlus-E1133	7.884888089	0.005536489
146196	hsa-miR-711	3.670788561	0.03965173
11091	hsa-miR-377	2.111223512	0.04976774
46580	hsa-miRPlus-E1077	5.288463894	0.000382255
46345	hsa-miR-1207-3p	2.379708256	0.039776182
145843	hsa-miR-330-5p	0.42740941	0.029114475
42865	hsa-miR-181a	0.209028098	0.009485987
10972	hsa-miR-181b	0.241496397	0.029192032
10937	hsa-miR-132	0.42440407	0.012416679
42654	hsa-miR-483-5p	0.302004694	0.019810712
145941	hsa-miRPlus-F1086	0.265815909	0.017106349
46755	hsa-miRPlus-E1026	0.110932379	0.010144043

Supplementary Table S2: Primers used for qRT-PCR

RASSF1A	5′-TCTGGGGCGTCGTGCGCAAA-3′
	5′-TCTGGGGCGTCGTGCGCAAA-3′
β-actin	5′-ACCCAGATCATGTTTGAGACC-3′
	5′-GGAGTTGAAGGTAGTTTCGTG-3′
U6	5′-GCTTCGGCAGCACATATACTAAAAT-3′
	5′-CGCTTCACGAATTTGCGTGTCAT-3′
hsa-miR-711 primer GSP:	5′-GGGACCCAGGGAGAGA-3′
hsa-miR-711 primer R:	5′-CAGTGCGTGTCGTGGA-3′
